# Supplementary material for: National Antibiotics Utilization Trends for Human Use in Tanzania from 2010 to 2016 Inferred from Tanzania Medicines and Medical Devices Authority Importation Data
Source: Antibiotics (Basel). 2021 Oct 15;10(10):1249. doi: 10.3390/antibiotics10101249 (PMC8532727; doi:10.3390/antibiotics10101249)
Supplement: Supplementary file 1 [file antibiotics-10-01249-s001.zip › antibiotics-1355349-supplementary.pdf]

## Supplementary Material

**Supplementary Table S1: Population estimates for Tanzania 2010 – 2017 according to the Tanzania Bureau of Statistics (TBS)**

| Year | Estimated population |
|---|---|
| 2010 | 46,100,000 |
| 2011 | 47,570,000 |
| 2012 | 49,080,000 |
| 2013 | 50,640,000 |
| 2014 | 52,230,000 |
| 2015 | 53,880,000 |
| 2016 | 55,570,000 |

**Supplementary Table S2: Variation of Defined Daily Dose (DDD per 1000 inhabitants per day (DID) antibiotics per sector of purchase of antibiotics utilized in Tanzania from 2010-2016**

| | **Defined Daily Dose (DDD per 1000 inhabitants per day (DID)** | | |
|---|---|---|---|
| **Year** | **Public sector** | **Private sector** | **Grand Total** |
| 2010 | 0.315695124 | 6.462989723 | 6.778684846 |
| 2011 | 3.264509044 | 9.998647410 | 13.26315645 |
| 2012 | 1.250840438 | 8.530454799 | 9.781295237 |
| 2013 | 0.073671843 | 14.58051563 | 14.65418747 |
| 2014 | 2.017742880 | 27.84687869 | 29.86462157 |
| 2015 | 0.102752823 | 31.87673690 | 31.97948972 |
| 2016 | 2.613756117 | 45.57334786 | 48.18710398 |
| **Years total** | **9.638968269** | **144.869571** | **154.5085393** |
| % | **6.238469611** | **93.76153039** | **100** |

**Supplementary Table S3: Variation of Defined Daily Dose (DDD per 1000 inhabitants per day (DID) antibiotics per dosage form of antibiotics utilized in Tanzania from 2010-2016**

| Dosage form/ | Defined Daily Dose (DDD per 1000 inhabitants per day (DID) | | | | | | | |
|---|---|---|---|---|---|---|---|---|
| Year | 2010 | 2011 | 2012 | 2013 | 2014 | 2015 | 2016 | All year's total |
| Capsule | 4.14502603 | 5.309291455 | 5.106272557 | 6.00690412 | 22.15500286 | 27.07317189 | 27.67566794 | 97.47133686 |
| Tablet | 2.122573461 | 7.209185713 | 3.914566033 | 7.260900909 | 5.447574003 | 3.839556442 | 19.16005039 | 48.95440695 |
| Syrup | 0.28356336 | 0.529064846 | 0.419095874 | 0.533276424 | 0.98205372 | 0.869443006 | 0.966824426 | 4.583321657 |
| Injectable | 0.216595104 | 0.202455566 | 0.306573632 | 0.784661864 | 1.27099537 | 0.186970845 | 0.336174748 | 3.304427128 |
| Solution | 0.010744837 | 0.012516484 | 0.023937911 | 0.036285644 | 0.004262286 | 0.000001 | - | 0.08774784 |
| Powder | 0.000180271 | 0.000642391 | 0.01062064 | 0.031460592 | 0.004732801 | 0.009004053 | 0.029945636 | 0.086586383 |
| Intravenous Infusion | 0.000002 | - | 0.00022859 | 0.000697916 | 0.000001 | 0.001342812 | 0.018440836 | 0.020712461 |
| **Total** | **6.778684846** | **13.26315645** | **9.781295237** | **14.65418747** | **29.86462157** | **31.97948972** | **48.18710398** | **154.5085393** |

**Supplementary Table S4: Variation of amounts DIDs and kg of antivirals and antifungals utilized in Tanzania from 2010-2017**

| Rank | Medicine (ATC code level 5) | Defined Daily Dose (DDD) measurement units. Utilization was expressed in DDD per 1000 inhabitants per day (DID) | | | | | | | |
|---|---|---|---|---|---|---|---|---|---|
| | Year | 2010 | 2011 | 2012 | 2013 | 2014 | 2015 | 2016 | All years total |
| 1 | Amoxicillin (J01CA04) | 2.138265 | 2.732834 | 3.166120 | 2.906303 | 15.186938 | 8.460106 | 19.188346 | 53.778913 |
| 2 | Metronidazole (J01XD01) | 0.719509 | 5.105518 | 0.921516 | 1.307620 | 1.569014 | 0.994656 | 13.243172 | 23.861005 |
| 3 | Tetracycline (J01AA07) | 0.121011 | 0.349499 | 0.033493 | 0.211189 | 3.117376 | 14.126072 | 2.569880 | 20.528519 |
| 4 | Ciprofloxacin (J01MA02) | 0.642624 | 0.857677 | 1.637936 | 1.224426 | 1.279803 | 1.195216 | 2.427715 | 9.265398 |
| 5 | Cefalexin (J01DB01) | 0.021399 | 0.013309 | 0.019791 | 0.074673 | 1.470269 | 2.649437 | 2.693602 | 6.942480 |

| | | | | | | | | | |
|---|---|---|---|---|---|---|---|---|---|
| 6 | Doxycycline (J01AA02) | 0.445702 | 0.698575 | 0.581497 | 0.772933 | 1.464953 | 0.750373 | 2.161038 | 6.875071 |
| 7 | Tinidazole (J01XD02) | 0.206036 | 0.229824 | 0.344764 | 1.914793 | 0.625587 | 0.440550 | 1.477362 | 5.238916 |
| 8 | Ampicillin (J01CA01) | 0.882168 | 1.182723 | 0.457627 | 0.438006 | 0.361189 | 0.920659 | 0.233299 | 4.475671 |
| 9 | Erythromycin (J01FA01) | 0.220473 | 0.324990 | 0.510476 | 0.330956 | 0.787203 | 0.481507 | 0.953598 | 3.609203 |
| 10 | Ampicillin + cloxacillin (J01CR50) | 0.108795 | 0.164541 | 0.433184 | 0.365566 | 0.854357 | 0.345550 | 1.187669 | 3.459662 |
| 11 | Sulfamethoxazole + trimethoprim (J01EE01) | 0.114876 | 0.455251 | 0.009334 | 1.785802 | 0.005797 | 0.083260 | 0.001800 | 2.456121 |
| 12 | Cloxacillin (J01CF02) | 0.207602 | 0.321452 | 0.452528 | 1.282708 | 0.013342 | | 0.003698 | 2.281330 |
| 13 | Phenoxymethyl penicillin (J01CE02) | 0.011937 | 0.064516 | 0.186077 | 0.338393 | 0.460273 | 0.243257 | 0.557363 | 1.861816 |
| 14 | Procaine benzylpenicillin (J01CE09) | 0.132676 | 0.120228 | 0.083775 | 0.256539 | 0.531735 | 0.046984 | 0.126253 | 1.298190 |
| 15 | Amoxicillin + clavulanate (J01CR02) | 0.082356 | 0.100458 | 0.140504 | 0.142044 | 0.232627 | 0.349376 | 0.231817 | 1.279183 |
| 16 | Azithromycin (J01FA10) | 0.094503 | 0.108884 | 0.114337 | 0.178926 | 0.246168 | 0.076106 | 0.179319 | 0.998243 |
| 17 | Benzathine penicillin (J01CE08) | 0.038184 | 0.041170 | 0.073368 | 0.121730 | 0.425541 | 0.078943 | 0.105260 | 0.884196 |
| 18 | Ampicillin + cloxacillin (J01CR50) | 0.044223 | 0.048281 | 0.065898 | 0.090380 | 0.143910 | 0.129011 | 0.154844 | 0.676548 |
| 19 | Levofloxacin (J01MA12) | 0.034375 | 0.038027 | 0.111927 | 0.136712 | 0.187886 | | | 0.508927 |
| 20 | Chloramphenicol (J01BA01) | 0.028010 | 0.023766 | 0.089806 | 0.144607 | 0.100552 | 0.050258 | 0.069742 | 0.506741 |
| 21 | Benzyl penicillin (J01CE01) | 0.003043 | 0.018718 | 0.060511 | 0.087510 | 0.092412 | 0.055496 | 0.093715 | 0.411406 |
| 22 | Gentamycin (J01GB03) | 0.005098 | 0.001082 | 0.019909 | 0.152425 | 0.199343 | 0.000167 | 0.023951 | 0.401975 |
| 23 | Ofloxacin (J01MA01) | 0.192910 | 0.035529 | 0.012030 | 0.021427 | 0.043066 | 0.074941 | 0.014443 | 0.394346 |
| 24 | Amoxicillin + flucloxacillin (J01CR50) | 0.020743 | 0.053618 | 0.044492 | 0.050579 | 0.060377 | 0.084763 | 0.054888 | 0.369460 |
| 25 | Clarithromycin + lansoprazole + tinidazole (A02BD09) | 0.017254 | 0.021855 | 0.037003 | 0.033176 | 0.048569 | 0.045817 | 0.072146 | 0.275821 |
| 26 | Cefuroxime (J01DC02) | 0.015055 | 0.021172 | 0.017329 | 0.051782 | 0.036431 | 0.071949 | 0.042467 | 0.256185 |
| 27 | Nitrofurantoin (J01XE01) | 0.000743 | 0.000014 | | 0.005453 | 0.104910 | 0.006407 | 0.108366 | 0.225894 |
| 28 | Clarithromycin (J01FA09) | 0.011396 | 0.021169 | 0.018348 | 0.023908 | 0.046976 | 0.051667 | 0.041063 | 0.214527 |
| 29 | Cefadroxil (J01DB05) | 0.022564 | 0.004272 | 0.008776 | 0.015943 | 0.037273 | 0.044224 | 0.028872 | 0.161923 |

| | | | | | | | | | |
|---|---|---|---|---|---|---|---|---|---|
| 30 | Ciprofloxacin + tinidazole (J01RA11) | 0.020266 | 0.018430 | 0.020166 | 0.032732 | 0.032391 | 0.008771 | 0.013874 | 0.146629 |
| 31 | Ornidazole (J01XD03) | 0.012338 | 0.016220 | 0.016459 | 0.018232 | 0.022623 | 0.019889 | 0.035764 | 0.141526 |
| 32 | Cefixime (J01DD08) | 0.007226 | 0.011580 | 0.018225 | 0.018968 | 0.020195 | 0.015190 | 0.021848 | 0.113232 |
| 33 | Clindamycin (J01FF01) | 0.083110 | 0.001855 | 0.001134 | 0.003860 | 0.006826 | 0.004883 | 0.002234 | 0.103901 |
| 34 | Cefpodoxime (J01DD13) | 0.003836 | 0.003133 | 0.008052 | 0.008979 | 0.020799 | 0.032830 | 0.024726 | 0.102355 |
| 35 | Ceftriaxone (J01DD04) | 0.011348 | 0.002159 | 0.022192 | 0.038954 | 0.000017 | 0.000008 | 0.000080 | 0.074759 |
| 36 | Norfloxacin (J01MA06) | 0.009422 | 0.004034 | 0.007918 | 0.010087 | 0.018146 | 0.011025 | 0.012003 | 0.072634 |
| 37 | Ceftazidime (J01DD02) | 0.000004 | 0.000005 | 0.014425 | 0.035810 | 0.000026 | 0.000138 | 0.000004 | 0.050413 |
| 38 | Roxithromycin (J01FA06) | | 0.036802 | 0.003014 | | | 0.002792 | | 0.042608 |
| 39 | Flucloxacillin (J01CF05) | 0.001070 | 0.001885 | 0.000317 | 0.000177 | 0.000197 | 0.013781 | 0.012665 | 0.030091 |
| 40 | Perfloxacin (J01MA03) | 0.026149 | | | | | | | 0.026149 |
| 41 | Sparfloxacin (J01MA09) | 0.012622 | | 0.010449 | | | | | 0.023070 |
| 42 | Nalidixic acid (J01MB02) | 0.002309 | 0.002607 | 0.002409 | 0.004045 | 0.001915 | 0.005148 | 0.003507 | 0.021939 |
| 43 | Cefaclor (J01DC04) | 0.003584 | 0.003053 | | 0.001601 | 0.001567 | 0.001623 | 0.003777 | 0.015204 |
| 44 | Linezolid (J01XX08) | | | | 0.009671 | 0.001705 | | 0.001104 | 0.012480 |
| 45 | Cefotaxime (J01DD01) | 0.000380 | 0.000316 | 0.000345 | 0.000257 | 0.000432 | 0.001872 | 0.004084 | 0.007687 |
| 46 | Cefprozil (J01DC10) | 0.000392 | 0.000317 | 0.000614 | 0.002040 | 0.000452 | 0.000647 | 0.001183 | 0.005646 |
| 47 | Sultamicillin (J01CR04) | 0.000605 | 0.000475 | 0.000544 | 0.000598 | 0.000939 | 0.000140 | 0.000732 | 0.004032 |
| 48 | Meropenem (J01DH02) | 0.000049 | 0.000070 | 0.000111 | 0.000183 | 0.000513 | 0.001954 | 0.000919 | 0.003800 |
| 49 | Cefepime (J01DE01) | 0.000309 | 0.000204 | 0.001016 | 0.000670 | 0.000272 | 0.000292 | 0.000200 | 0.002962 |
| 50 | Amikacin (J01GB06) | | 0.000254 | 0.000746 | 0.000284 | | 0.000381 | 0.000185 | 0.001851 |
| 51 | Oxytetracycline combinations (J01AA56 ) | | 0.000376 | | | 0.001385 | | | 0.001761 |
| 52 | Moxifloxacin (J01MA14) | 0.000091 | 0.000101 | 0.000095 | 0.000082 | 0.000001 | | 0.000828 | 0.001197 |
| 53 | Ceftriaxone combinations (J01DD54) | | | | | | 0.000172 | 0.000961 | 0.001133 |
| 54 | Piperacillin + tazobactam (J01CR05) | 0.000043 | 0.000037 | 0.000205 | 0.000280 | 0.000117 | 0.000061 | 0.000079 | 0.000822 |
| 55 | Cefoperazone, combinations (J01DD62) | | | | | 0.000066 | 0.000443 | 0.000243 | 0.000752 |
| 56 | Vancomycin (J01XA01) | | 0.000069 | 0.000165 | 0.000051 | 0.000122 | 0.000064 | 0.000160 | 0.000630 |
| 57 | Streptomycin (J01GA01) | 0.000000 | 0.000096 | 0.000237 | | 0.000001 | 0.000001 | 0.000135 | 0.000469 |

| | | | | | | | | | |
|---|---|---|---|---|---|---|---|---|---|
| 58 | Ampicillin combination (J01CA51) | | | 0.000006 | 0.000020 | 0.000007 | 0.000381 | | 0.000415 |
| 59 | Cefazolin (J01DB04) | | 0.000000 | | 0.000079 | 0.000022 | 0.000251 | | 0.000353 |
| 60 | Chlortetracycline (J01AA03) | | 0.000125 | 0.000089 | | | | | 0.000214 |
| 61 | Trimethoprim (J01EA01) | | | | | | | 0.000119 | 0.000119 |
| 62 | Ampicillin + sulbactam (J01CR01) | | | 0.000006 | 0.000020 | 0.000006 | 0.000004 | | 0.000037 |
| 63 | Spiramycin + metronidazole (J01RA04) | | | | | 0.000000 | | | 0.000000 |

**Supplementary Table S5: The autoregressive integrated moving average (ARIMA) (0, 1, 0) model predicts the significant increase in utilization and forecasts the trends of antibiotics up to the period 2022 modeled using the data from 2010-2016. The model estimated that by 2022, the total of antibiotics consumed would reach 89.60 DIDs**

| Year | Predicted total DIDs of consumed antibiotics | Lower confidence limits | Upper confidence limits |
|---|---|---|---|
| 2010 | | | |
| 2011 | 13.68 | -5.92 | 33.28 |
| 2012 | 20.16 | 0.56 | 39.76 |
| 2013 | 16.68 | -2.92 | 36.28 |
| 2014 | 21.55 | 1.95 | 41.15 |
| 2015 | 36.76 | 17.16 | 56.36 |
| 2016 | 38.88 | 19.28 | 58.48 |
| 2017 | 55.09 | 35.49 | 74.69 |
| 2018 | 61.99 | 34.28 | 89.71 |
| 2019 | 68.9 | 34.95 | 102.84 |
| 2020 | 75.8 | 36.6 | 114.99 |
| 2021 | 82.7 | 38.88 | 126.52 |
| 2022 | 89.6 | 41.59 | 137.61 |
